# Glomerular Basement Membrane Selective Permeability in Short-term Streptozotocin-induced Diabetic Rats

**DOI:** 10.1155/EDR.2000.19

**Published:** 2000

**Authors:** Michele Doucet, Irene Londoño, Amparo Gómez-Pascual, Moise Bendayan

**Affiliations:** 1 Department of Pathology and Cell Biology Université de Montréal Montreal Québec H3C 3J7 Canada; 2 Anatomo-Pathology Universidad de Sevilla Sevilla Spain; 3 Département de Pathologie et Biologie Cellulaire Université de Montréal Succursale Centre-ville C.P. 6128 Montréal Québec H3C 3J7 Canada

## Abstract

In diabetes, the glomerular basement membrane undergoes thickening and structural alterations with loss of glomerular permselectivity properties. However, the onset of the alterations at early phases of diabetes is unclear. Aiming to determine the functional and structural alterations of the glomerular wall in the early stages of diabetes, we have studied the distribution of endogenous circulating albumin and type IV collagen in the glomerular basement membrane, using the immunocytochemical approach. The streptozotocin-injected hyperglycemic rat was our animal model. Renal tissues were examined after 10 days, 2, 4 and 6 months of hyperglycemia. Upon immunogold labelings, changes in the glomerular permeability to endogenous albumin were found altered as early as upon ten days of hyperglycemia. In contrast, no structural modifications were detected at this time point. Indeed, glomerular basement membrane thickening and an altered type IV collagen labeling distribution were only observed after four months of hyperglycemia, suggesting that functional alterations take place early in diabetes prior to any structural modification. In order to evaluate the reversibility of the glomerular alterations, two-month-old diabetic animals were treated with insulin. These animals showed a significant restoring of their glomerular permselectivity. Our results suggest a link between glycemic levels and alteration of glomerular permeability in early stages of diabetes, probably through high levels of glycated serum proteins.


*Int. Jnl. Experimental Diab. Res.*, Vol. 1, pp. 19–30
Reprints available directly from the publisher
Photocopying permitted by license only

© 2000 OPA (Overseas Publishers Association) N.V.
Published by license under
the Harwood Academic Publishers imprint,
part of The Gordon and Breach Publishing Group.
Printed in Malaysia.

### Glomerular Basement Membrane Selective Permeability in Short-term Streptozotocin-induced Diabetic Rats

MICHELE DOUCET, IRENE LONDOÑO, AMPARO GÓMEZ-PASCUAL* and MOISE BENDAYAN†

*Department of Pathology and Cell Biology, Université de Montréal, Montréal, Québec, Canada, H3C 3J7*

*(Received 15 May 1999; In final form 24 August 1999)*

**In diabetes, the glomerular basement membrane undergoes thickening and structural alterations with loss of glomerular permselectivity properties. However, the onset of the alterations at early phases of diabetes is unclear. Aiming to determine the functional and structural alterations of the glomerular wall in the early stages of diabetes, we have studied the distribution of endogenous circulating albumin and type IV collagen in the glomerular basement membrane, using the immunocytochemical approach. The streptozotocin-injected hyperglycemic rat was our animal model. Renal tissues were examined after 10 days, 2, 4 and 6 months of hyperglycemia. Upon immunogold labelings, changes in the glomerular permeability to endogenous albumin were found altered as early as upon ten days of hyperglycemia. In contrast, no structural modifications were detected at this time point. Indeed, glomerular basement membrane thickening and an altered type IV collagen labeling distribution were only observed after four months of hyperglycemia, suggesting that functional alterations take place early in diabetes prior to any structural modification. In order to evaluate the reversibility of the glomerular alterations, two-month-old diabetic animals were treated with insulin. These animals showed a significant restoring of their glomerular permselectivity. Our results suggest a link between glycemic levels and alteration of glomerular permeability in early stages of diabetes, probably through high levels of glycated serum proteins.**

*Keywords:* Glomerular basement membrane, albumin, type IV collagen, insulin, immunocytochemistry

### INTRODUCTION

The glomerular wall plays an important role in blood filtration. Its properties have been extensively studied by ultrastructural approaches using various circulating endogenous and exogenous markers (Farquhar *et al.*, 1961; Graham and Karnovsky, 1966; Caufield and Farquhar, 1974; Laliberté *et al.*, 1978; Bendayan *et al.*, 1986). Such studies have demonstrated that the transglomerular passage of serum proteins and macromolecules is highly selective. Serum albumin for example, which has a molecular weight of 68 kDa, is normally excluded from transglomerular transport and is not found in

*Present address: Anatomo-Pathology, Universidad de Sevilla, Sevilla, Spain.
†Corresponding author. Département de Pathologie et Biologie Cellulaire, Université de Montréal, C.P. 6128, Succursale Centre-ville, Montréal, Québec, Canada H3C 3J7. Tél.: (514) 343-6289, e-mail: moise.bendayan@umontreal.ca significant amounts in the urine (Bendayan *et al.*, 1986; Pappenheimer, 1953; Brenner *et al.*, 1978a; Ryan and Karnovsky, 1976). The exclusion of this protein has been considered to be the combined result of size restriction and electrostatic charge repulsion (Brenner *et al.*, 1978). However, it has been well documented that in diabetic nephropathy, the glomerular permselectivity is markedly impaired, leading to proteinuria (Hostetter, 1986; Viberti *et al.*, 1983). The loss of glomerular selective permeability occurring in a long-term diabetic condition appears to be concomitant with several other alterations such as increase in glomerular filtration rate (Ditzel and Junker, 1973; Chistiansen *et al.*, 1981), glycation of circulating proteins (Bunn *et al.*, 1978; Dolholfer and Weiland, 1980; McFarland *et al.*, 1979; Guthrow *et al.*, 1979; Day *et al.*, 1980) and basement membrane components (Cohen *et al.*, 1980; Trueb *et al.*, 1984; Garlick *et al.*, 1988; Bendayan, 1988), as well as modifications in the structure and composition of the basement membrane (Farquhar *et al.*, 1959; Hirose *et al.*, 1982; Bendayan, 1985; Østerby,1986; Desjardins and Bendayan, 1990; Beisswenger and Spiro, 1970; Pathasarathy and Spiro, 1982; van den Born *et al.*, 1995). Therefore, associating a single ethiopathological factor to the pathogenesis of glomerular permeability is rather difficult.

Several studies have investigated renal alterations occurring early in diabetes. Renal extracellular matrix gene expression in early stages of experimental diabetes was found to be altered with a marked increase in the mRNAs expression of $\alpha$ chains of type IV procollagen (Wu *et al.*, 1997; Fukui *et al.*, 1992; Yang *et al.*, 1995; Park *et al.*, 1997) and $\beta$ chains of laminin (Fukui *et al.*, 1992; Yang *et al.*, 1995). In addition, increases in expression of the inhibitor of metalloproteinase-1 (Wu *et al.*, 1997) and TGF-$\beta$ (Yang *et al.*, 1995; Park *et al.*, 1997) mRNAs have also been reported. In contrast, expression of heparan sulfate proteoglycans (Fukui *et al.*, 1992) and metalloproteinase-2 (Wu *et al.*, 1997) mRNAs seems to undergo decreases. Moriya *et al.*(1993) have demonstrated that changes in the charge selective barrier of the glomerular basement membrane (GBM) may occur in early stages of experimental diabetes, as early as one week after induction of diabetes. Physiological studies have, on the other hand, shown the existence of microalbuminuria prior to the development of the clinically persistent proteinuria in human diabetes (Chiarelli *et al.*, 1997). Microalbuminuria in early phases of diabetic nephropathy seems to be due to, or is concomitant with, disturbances of the charge barrier of the GBM and is followed by damages to the size restriction barrier (Dechert *et al.*, 1998; Viberti and Wiseman, 1986). Although streptozotocin-induced-diabetic rats are generally used for the study of the early phases of diabetic nephropathy, timing in the loss of selective permeability properties of the GBM in this experimental model has yet to be determined. The present study was focused on the glomerular wall, assessing its functional and structural features rather than the overall renal function through clearance evaluations which include the tubular reabsorption.

Application of the post-embedding immunogold method on renal tissues allows for the high-resolution localization of endogenous albumin within the glomerular wall. This approach was previously applied to the investigation of glomerular permeability in long-term diabetic animals and carries major advantages (Bendayan *et al.*, 1986). Indeed, the post-embedding approach allows for the detection of serum proteins retained *in situ* under physiological steady-state conditions. This contrasts with methods using exogenous electron-dense tracers introduced into the circulation with adverse effects on vascular permeability (Cotran and Karnovsky, 1967; Simionescu, 1979; Villaschi *et al.*, 1986). Furthermore, due to the particulate nature of the colloidal gold marker, morphometric evaluations can be performed assessing the passage of endogenous serum components through vascular walls (Bendayan *et al.*, 1986; Ghitescu and Bendayan, 1992; Bendayan and Rasio, 1996). In the present study, we have investigated the distribution of endogenous serum albumin across the glomerular wall, as a parameter reflecting the glomerular permselectivity properties. The work was carried out on animals at early stages of diabetes as well as on insulin-treated diabetic animals. We established correlations between the time points at which glomerular functional alterations and ultrastructural modifications appear as well as the reversibility of these alterations upon insulin treatment. Results have demonstrated that as early as upon ten days of hyperglycemia, albumin distribution through the glomerular basement membrane is altered, despite the absence of glomerular basement membrane thickening and normal type IV collagen distribution. In addition, glycemic control achieved by insulin treatment appears to restore the permselectivity properties of the glomerular wall in an early phase of diabetes.

### MATERIALS AND METHODS

### Animals

An experimental hyperglycemic state was induced in 100 g male Sprague-Dawley rats by a single intraperitoneal injection of streptozotocin (70 mg/kg body weight) dissolved in 10 mmol/l citrate buffer, pH 4.5. The hyperglycemic state developed within the first 48 h after injection and continued throughout the entire lifetime of the animals. This was assessed by regular measurements of glycosuria and glycemia carried out using Multistix and Dextrostix reagent strips (Miles Ames, Ontario, Canada). Levels of circulating insulin (postprandial) were determined by radioimmunoassay (insulin RIA kit, Chromacod, Bio Ria, Montreal, Quebec) on blood samples collected at the end of each experiment. Animals of the control group, which received only the citrate buffer, remained normoglycemic. Nine groups, each comprising at least three animals, were created. Groups 1 to 4 consisted of streptozotocin-injected animals of ten days, two, four and six months of hyperglycemia, respectively. Group 5 consisted of two-month hyperglycemic animals which afterwards were treated subcutaneously by daily injections of human biosynthetic insulin (Eli Lilly Co., Indianapolis, IN, USA) for one month. The amounts of insulin ranged from 15 to 20 units/day depending on the animal and were adjusted according to bi-weekly blood glucose monitoring. Groups 6, 7, 8 and 9 consisted of age-matched control animals to groups 1 to 4. At time of death, the body weight was measured and blood samples were taken. Streptozotocin-injected rats displayed high blood glucose and low insulin levels compared to the age-matched control animals (Tab. I). Blood glucose levels averaged $29.4 \pm 1.0$ mmol/l for the hyperglycemic animals (groups 1 to 4) *vs.* $6.6 \pm 0.35$ mmol/l for their age-matched controls (groups 6 to 9) and $4.9 \pm 0.8$ mmol/l for the insulin-treated streptozotocin-injected animals (group 5). Levels of circulating glycated albumin were measured by radioimmunoassay (Exocell Inc., Philadelphia, PA). Ten days hyperglycemic animals display about 3.5 times more glycated albumin than their age-matched controls ($1.46 \pm 0.07$ mg/ml *vs.* $0.41 \pm 0.02$ mg/ml for the controls), while total serum albumin did not show any significant variation ($33.95 \pm 0.40$ mg/ml *vs.* $36.66 \pm 3.10$ mg/ml for the controls). The hyperglycemic animals also displayed important polyuria with total urine 24 h volumes of $113 \pm 13$ ml (*vs.* 6.7+1.5 ml for the controls). Protein and albumin excretion were of $70.8 \pm 8.6$ mg/24 h and $21.5 \pm 6.0$ mg/24 h respectively (*vs.* $38.9 \pm 7.0$ mg/24 h and $1.5 \pm 0.5$ mg/24 h for the controls).

### Tissue Processing

The animals were anesthetized with urethane and the kidney cortex was immediately fixed *in situ* by immersion with a periodate-lysine-paraformaldehyde solution (McLean *et al.*, 1974). Small tissue fragments were sampled and maintained in the fixative for two hours at 4°C. They were th rinsed in 0.1 mol/l phosphate

TABLE I Characteristics of the control and diabetic rats

| Length of time | | Weight (g) | Plasma glucose levels(mmol/l) | Plasma insulin levels(μU/ml) | GBM thickness(nm) |
|---|---|---|---|---|---|
| 10 days | control | 160 ± 2 | 6.9 ± 0.61 | 28.0 ± 4.0 | 240 ± 39 |
| | diabetic | 148 ± 2 | 26.7 ± 1.2† | 29.0 ± 0.5 | 240 ± 17 |
| 2 months | control | 405 ± 5 | 7.5 ± 0.25 | 66.0 ± 11.0 | 230 ± 23 |
| | diabetic | 286 ± 26† | 30.7 ± 0.7† | 32.0 ± 3.0† | 222 ± 35 |
| 4 months | control | 566 ± 17 | 6.0 ± 0.2 | 43.5 ± 7.0 | 220 ± 35 |
| | diabetic | 290 ± 25† | 28.9 ± 1.2† | 31.5 ± 7.0† | 260 ± 35* |
| 6 months | control | 750 ± 25 | 6.1 ± 0.89 | 30.9 ± 3.3 | 270 ± 28 |
| | diabetic | 187 ± 17† | 31.2 ± 1.2† | 18.5 ± 0.5† | 320 ± 35* |

mean values ± s.e.m., $n = 3-5$ animals; $^{*}p < 0.05$; $^{\dagger}p < 0.001$.

buffer, dehydrated in methanol and embedded in Lowicryl K4M at − 20°C as previously described (Bendayan, 1995). Ultrathin sections were cut, mounted on Parlodion-carbon coated nickel grids and processed for immunocytochemistry.

### Immunocytochemistry

Antigenic sites for endogenous albumin and type IV collagen were revealed using specific polyclonal antibodies (Cappel, West Chester, PA, USA and Chemicon, Temecula, CA, USA, respectively) in combination with the protein A-gold post-embedding immunocytochemical technique. The labeling procedure was carried out as previously described (Bendayan, 1995). Briefly, the grids carrying the tissue thin-sections were incubated on a drop of 0.15 mol/l glycine in 0.01 mol/l phosphate buffered saline pH 7.3 (PBS) for 20 min, and transferred on a drop of PBS containing 1% gelatin (Nanoprobes Inc, NY, USA) for an additional 10 min. Next, the grids were incubated overnight at 4°C on a drop of the anti-albumin polyclonal antibody (1 : 50) or 3 hours at room temperature on a drop of the anti-type IV collagen polyclonal antibody (1 : 40). After rinsing in PBS, the sections were incubated with 0.15 mol/l glycine in PBS for 20 min and transferred on a drop of the protein A-gold complex (10 or 15 nm) for a 30 min incubation at room temperature (Bendayan, 1995). The grids were then washed with PBS, rinsed in distilled water and dried. The tissue sections were stained with uranyl acetate and examined with a Philips 410 electron microscope. The specificity of each immunolabeling was assessed by various control experiments: incubation with the protein A-gold solution alone, omitting the antibody step, and incubation with the antibody solution to which the corresponding antigen was added in excess, followed by the protein A-gold complex.

### Morphometric Analysis

For both antigens, the exact location of the gold particles over the glomerular basement membrane was analyzed by a morphometrical approach using an image processing system (Videoplan 2, Carl Zeiss Inc., Toronto, Canada). The pictures were recorded at × 16 900 and printed to a final magnification of × 42 250. At least fifteen pictures were analyzed for each antigen and for each animal. The distance between each individual gold particle and the abluminal plasma membrane at the base of the endothelial cells was recorded. Simultaneously, at the same site, the distance between the base of endothelial cell and the podocyte was also measured. The ratio $R$ = [distance (Endothelium − gold particle)/distance (Endothelium − Epithelium)] between these two values was calculated. On average, 500 ratio values were recorded for each animal and each antigen. The distribution of these ratio values ($0 < R < 1$) was determined and represented as histograms.

Mean values were calculated and statistically evaluated using the Mann-Whitney U test.

### RESULTS

Electron microscopy examination of renal tissue revealed the glomerular basement membrane between the endothelial and the epithelial cells of the glomerular wall, with its three characteristic regions: the lamina lucida interna on the subendothelial side, the lamina densa in the central part and the lamina lucida externa on the epithelial side (Fig. 1). Morphometrical analysis showed that basement membrane thickness of control and hyperglycemic animals at ten days and two months is similar. In contrast, the four-and six-month hyperglycemic animals

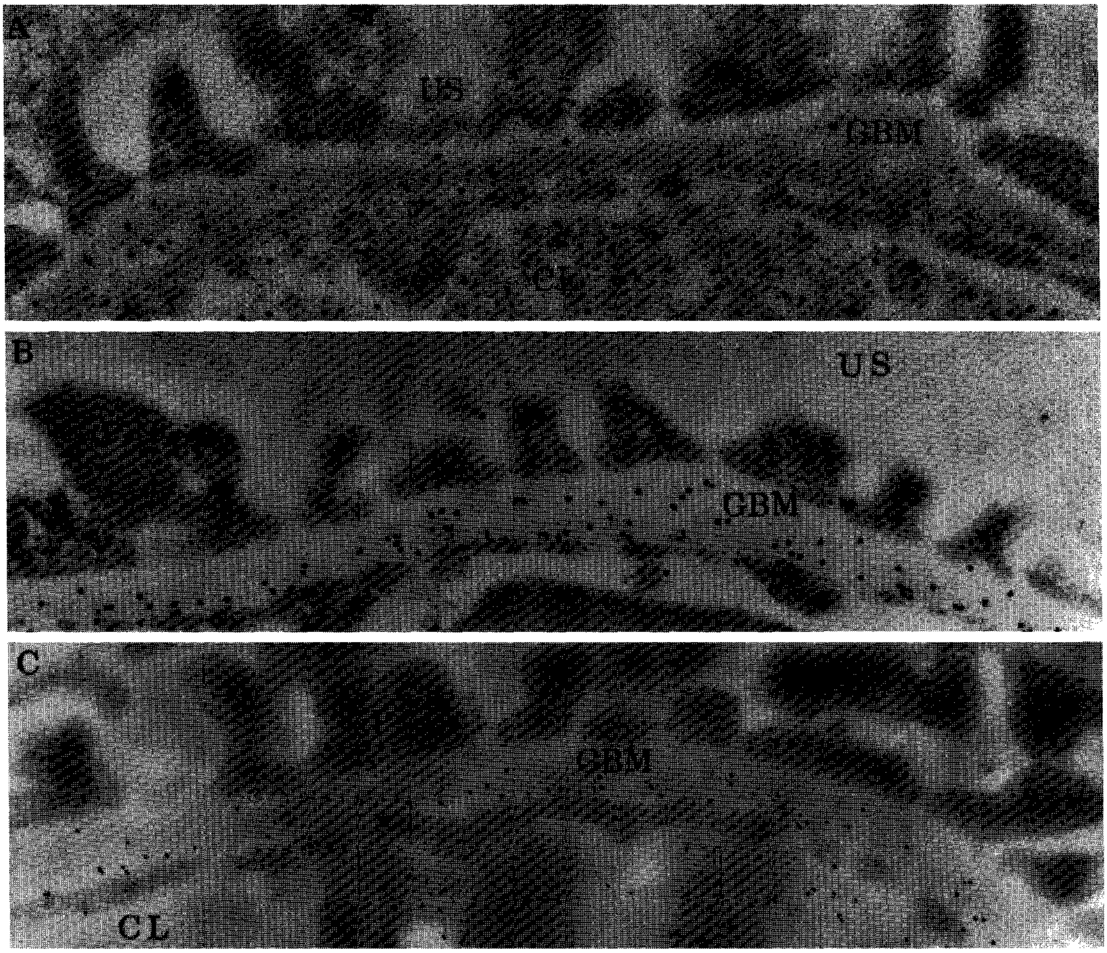

FIGURE 1 **Immuno-electron microscopy of endogenous albumin.**
(A) Renal tissue of a two-month-old normoglycemic animal. The glomerular wall appears normal with a thin glomerular basement membrane (GBM). The immunolabeling by gold particles, revealing albumin antigenic sites over the GBM, is mainly located over the subendothelial side. Flocculent material present in the capillary lumen (CL) is also labeled. The urinary space (US) as well as podocytes (P) are almost free of labeling; (B) Renal tissue of a two-month old hyperglycemic animal. The glomerular wall shows no significant morphological changes. GBM displays a normal thickness. Gold particles are present over the subendothelial as well as the central and subepithelial side of the GBM; (C) Renal tissue of a two-month-old streptozotocin-injected insulin-treated animal. The labeling by gold particles is located mainly over the subendothelial side of the GBM. (A × 50 000; B × 44 500; C × 49 000).

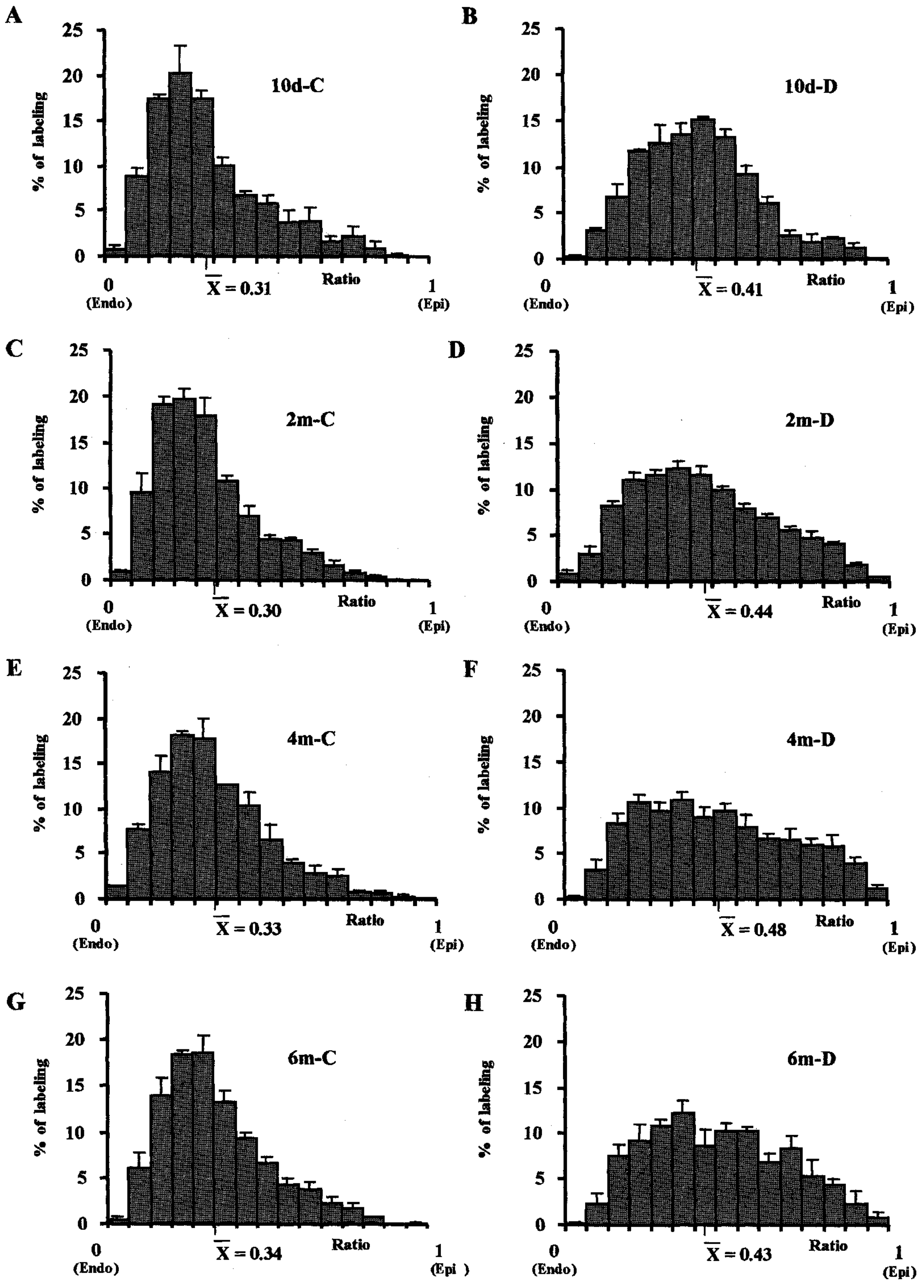

FIGURE 2 **Morphometrical analysis of albumin distribution.**

Distribution of rat albumin immunolabelings in the glomerular basement membrane of normoglycemic and streptozotocin-injected hyperglycemic animals as expressed in ratio values $R$ = [distance (Endothelium – gold particle) / distance (Endothelium – Epithelium)]. The histograms of all control animals (A,C,E,G) show asymmetrical distributions of the labeling, with peaks in the region corresponding to the subendothelial side of the glomerular basement membrane. For the streptozotocin-injected hyperglycemic animals (B,D,F,H), the distributions of the labeling appear significantly shifted to the right.

display significant thickenings with respect to the normoglycemic age-matched animals (Tab. I). In addition, at six months in hyperglycemic condition, the mesangial regions display large accumulations of basement membrane material, confirming previous reports (Bendayan *et al.*, 1986; Farquhar *et al.*, 1959; Hirose *et al.*, 1982; Østerby, 1986).

In normoglycemic animals, gold particles revealing endogenous rat albumin antigenic sites were found over the flocculent material present in the capillary lumina (circulating protein), as well as over the glomerular basement membrane. Labeling was almost absent in the urinary space. The labeling over the glomerular basement membrane appeared intense over the subendothelial side with few particles over the lamina densa. The lamina lucida externa on the epithelial side was almost free of labeling (Fig. 1A). In tissues from hyperglycemic animals, the distribution of the labeling was similar to that of the normoglycemic animals although the gold particles were more numerous over the lamina densa and lamina lucida externa (Fig. 1B). Intense labeling was also detected over the enlarged mesangial regions. Treatment of hyperglycemic animals with insulin appeared to reverse somehow the distribution of this labeling, the gold particles being more numerous over the lamina lucida interna (Fig. 1C).

Morphometrical evaluation of the distribution of the labelings through the GBM confirmed these subjective observations. The ratio values "$R$" between the distance of the gold particles to the abluminal endothelial plasma membrane and the thickness of the basement membrane at the same site, generated on tissues from normal and hyperglycemic animals, are reported in Figure 2. In the normoglycemic animals, a peak was systematically found at the level of the subendothelial side of the GBM ($0.30 < R < 0.34$) (Figs. 2A, 2C, 2E, 2G). In contrast, hyperglycemic animals displayed much more extended distributions with the major peak shifting to the right towards the subepithelial side of the GBM ($R > 0.4$) (Figs. 2B, 2D, 2F, 2H). The shifts occur as early as upon ten days of hyperglycemia and become more important along with the duration of the hyperglycemic state. The Mann–Whitney U test demonstrated the significance of the differences between labeling distributions from normoglycemic and hyperglycemic animals at time points as early as after ten days of hyperglycemia and thereafter ($p < 0.01$). On the other hand, the labeling distribution of albumin for the insulin-treated streptozotocin-injected

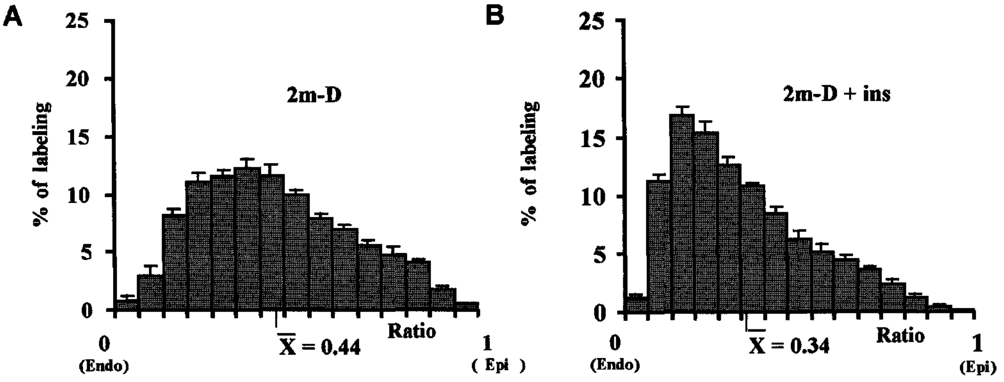

FIGURE 3 **Morphometrical analysis of albumin distribution.**
Distribution of rat albumin immunolabeling in the glomerular basement membrane of streptozotocin-injected hyperglycemic animals (3A) and streptozotocin-injected insulin-treated animals (3B) as expressed in ratio values ($R$). The histogram of the streptozotocin-injected insulin-treated animals (3B) is similar to that of the normoglycemic animals (2C) but differs significantly from that of the age-matched hyperglycemic animals (3A).

animals (Fig. 3B) was similar to that of the age-matched normoglycemic animals (Fig. 2C) and differed significantly from that of the age-matched hyperglycemic animals ($p < 0.01$) (Fig. 3A).

The ultrastructural localization of type IVcollagen was also studied on tissues of two-and four-month hyperglycemic and age-matched control animals. Labeling by gold particles was restricted to the extracellular matrix and was absent from the capillary lumina, urinary space and glomerular cells (Fig. 4). The labeling of the glomerular basement membrane was mainly located over the lamina densa in tissues of two-month normoglycemic and hyperglycemic animals (Fig. 4). At four months of hyperglycemia, the labeling was distributed mainly on the subendothelial side of the GBM which indicates

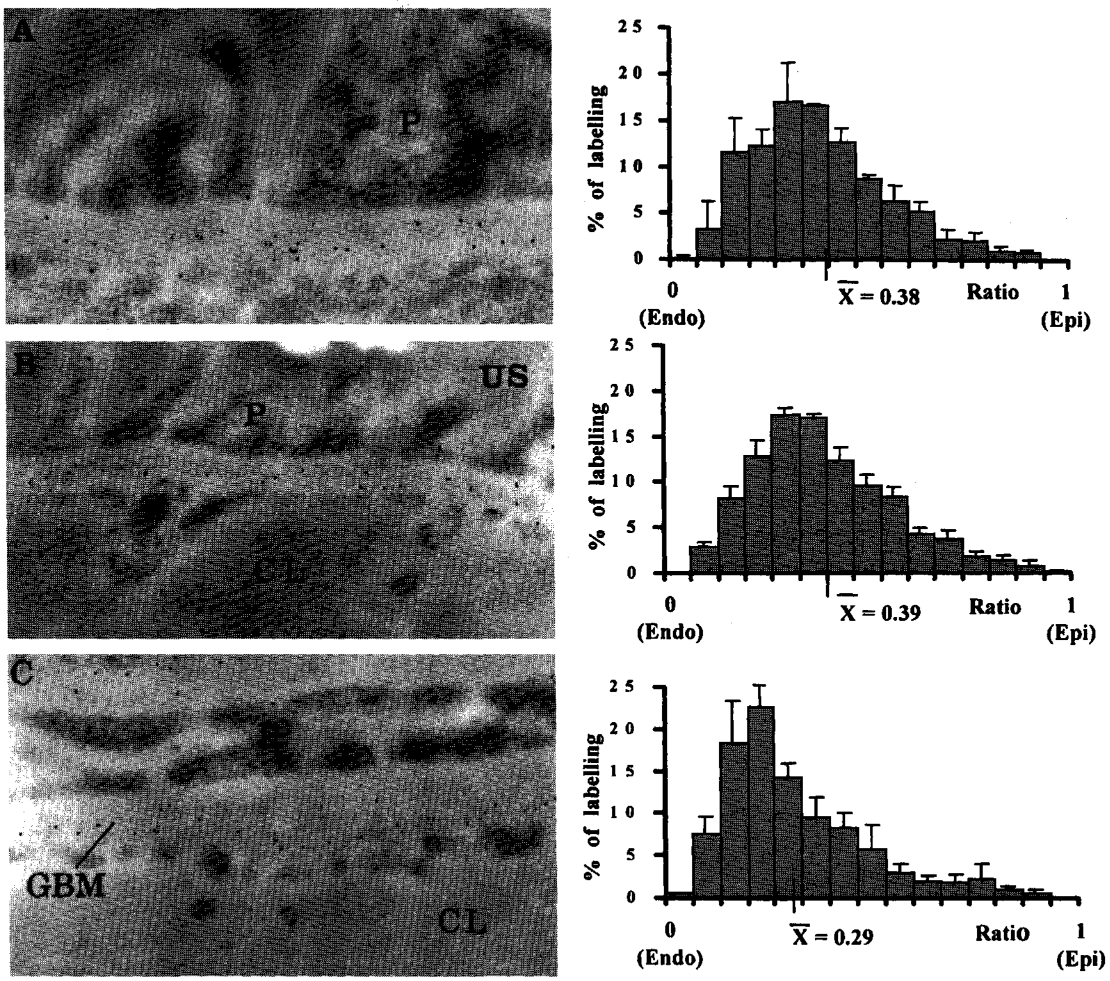

FIGURE 4 **Immuno-electron microscopy and morphometric analysis of type IV collagen.**
Immunolabelings for type IV collagen in the glomerular basement membrane of two-month normoglycemic (A) and two- (B) and four-month (C) streptozotocin-injected hyperglycemic animals. Labeling by gold particles is concentrated on the central lamina densa for the normoglycemic and hyperglycemic animals up to two months. In contrast, tissues from the four-month hyperglycemic animals display significant changes with labelings located towards the subendothelial side of the GBM. The distribution of the labeling, as expressed in ratio (*R*) values, reported in corresponding histograms, demonstrate significant changes only in tissues of four-month hyperglycemic animals. (A × 39 000; B × 32 000; C × 30 000).

an important alteration in the type IV collagen distribution. The morphometrical evaluation reported in Figure 4 confirmed these results.

The experiments performed to assess the specificity of the immunocytochemical results yield either no labeling or few gold particles over the different glomerular structures (results not illustrated).

### DISCUSSION

In the present study, the protein A-gold immunocytochemical technique was applied on renal tissues of normal and hyperglycemic animals as well as of insulin-treated animals for assessing the passage of endogenous albumin through the glomerular wall. In contrast to the clearance studies which evaluate the overall GBM filtration and tubular reabsorption (Hardwicke and Squire, 1955), the morpho-immunocytochemical study allows for the evaluation of processes occurring solely at the level of the glomerular wall (Bendayan *et al.*, 1986; Ryan and Karnovsky, 1976). Streptozotocin-injected hyperglycemic animals displayed the typical alterations related to diabetes with high blood glucose and low insulin levels with strong proteinuria and major deficiency in weight gain. In addition, animals with four or more months of hyperglycemia displayed the renal alterations characteristic of the diabetic glomerulopathy with thickening of glomerular basement membrane and enlargement of mesangial matrix (Hostetter, 1986; Farquhar *et al.*, 1959; Østerby, 1986).

In the normoglycemic animals of all ages, albumin antigenic sites were detected in capillary lumina and in the GBM mainly on its subendothelial side, few being located to the epithelial side. This suggests that in normal condition, albumin is mainly retained by the GBM preventing its passage from blood to the urinary space. This result is in agreement with previously reported ones (Bendayan *et al.*, 1986; Ryan and Karnovsky, 1976). In contrast, in hyperglycemic animals, including those at ten days, albumin antigenic sites were no longer preferentially accumulated on the subendothelial side of the GBM, the distribution of the labeling having shifted towards the epithelial side of the GBM. This indicates that the retention of albumin by the GBM is altered in short-term hyperglycemic condition, as it is for long-term diabetic animals (Bendayan *et al.*, 1986). As a matter of fact, many studies have established that important alterations in the biochemical and biophysical properties of the GBM occur in long-term diabetes and follow, or are concomitant with, the loss of the glomerular permselectivity. These alterations correspond mostly to the thickening of the GBM which constitutes the hallmark of diabetic glomerulopathy (Østerby, 1986; Schleicher and Olgemöller, 1992) and is the result of enhanced synthesis and decreased turnover of its structural components (Cohen *et al.*, 1982). Although recent observations reported increased expression of TGF-$\beta$ and type IV procollagen (Wu *et al.*, 1997; Fukui *et al.*, 1992; Yang *et al.*, 1995; Park *et al.*, 1997) and a decreased expression of metalloproteinases (Wu *et al.*, 1997; Suzuki *et al.*, 1997) in the GBM at the early phase of diabetes, the accumulation of GBM components as early as upon three months of diabetes is controversial (Park *et al.*, 1997). In our study, GBM thickening was observed only after four months of hyperglycemia. Along with this thickening, changes in the distribution of type IV collagen were also observed after four months of hyperglycemia as in long-term diabetes (Bendayan, 1985; Østerby, 1986; Desjardins and Bendayan, 1990; Inoue and Bendayan, 1995). These structural alterations thus appear long after changes in glomerular permselectivity to albumin which occur as early as upon ten days of hyperglycemia, indicating that the onset of morphological changes does not correlate with the early loss of GBM selective filtration properties. The possibility that such early alterations could in principle be attributed to a direct nephrotoxic action of streptozotocin can be ruled out since the alterations of the glomerular wall observed in the hyperglycemic animals are fully restored by the insulin treatment.

Loss of glomerular filtration properties in early diabetes could be explained by other factors or combination of factors. Heparan sulfate proteoglycans (HSPG), through their anionic charges, play important roles in the filtration properties of the GBM (Pathasarathy and Spiro, 1982; van den Born *et al.*, 1995). Decrease synthesis of glomerular HSPG was previously found in early (Moriya *et al.*, 1993; Rohrbabach *et al.*, 1983) as well as late (Kanwar *et al.*, 1983) stages of experimental diabetes. Therefore, charge barrier alterations could explain the early loss of permselectivity properties of the GBM (Moriya *et al.*, 1993). On the other hand, physiological factors such as the glomerular plasma flow and filtration rates also affect glomerular function (Hostetter *et al.*, 1981; Anderson and Brenner, 1988), but their real contribution to the loss of glomerular permselectivity in early stages of diabetes remains, however, uncertain (Castellino *et al.*, 1990).

Recent studies indicate that alterations in vascular permeability during diabetes could be due to glycation of serum proteins (Williams *et al.*, 1981; Wieland, 1983; Sampietro *et al.*, 1987; Daniels and Hausser, 1992; Sabbatini *et al.*, 1992; Londoño *et al.*, 1995; Bendayan and Londoño, 1996; Cohen and Ziyadeh, 1994). Amadori products are the predominant forms of circulating glycated proteins in diabetic subjects (Ziyadeh and Cohen, 1993). Their formation is rapid (Day *et al.*, 1980; Cohen and Ziyadeh, 1994; Higgins and Bunn, 1981) and their half-life short (Shima *et al.*, 1991). Therefore, levels of Amadori products in serum are directly related to the ambient glucose and are significantly enhanced in diabetic conditions (Neuman *et al.*, 1994; Bakala *et al.*, 1995). *In vitro* (Williams *et al.*, 1981; Ghiggeri *et al.*, 1984; Shaklai *et al.*, 1984) and *in vivo* (Sampietro *et al.*, 1987; Sabbatini *et al.*, 1992; Ghiggeri *et al.*, 1984; Layton and Jerums, 1988) studies have demonstrated that Amadori products increase vascular permeability. Along the same line, *in vitro* studies on isolated glomerular basement membranes suggest that the sieving coefficient for glycated albumin is greater than that for the native one (Daniels and Hausser, 1992). Recent studies have also shown that glycated albumin injected into the circulation of normal animals easily crosses the glomerular wall and increases the filtration of non-glycated albumin (Londoño *et al.*, 1995; Bendayan and Londoño, 1996). Alteration of albumin conformation as well as particular interactions with GBM components have been proposed to explain this enhanced glomerular passage of native albumin (Londoño *et al.*, 1995; Ghiggeri *et al.*, 1984; Layton and Jerums, 1988).

Our present results also show that the altered glomerular albumin distribution in two-month hyperglycemic animals is restored when blood glucose levels are normalized by insulin treatment. This supports the assumption that insulin treatment can restore permselective properties of the GBM. Since Amadori adducts formation is directly related to blood glucose levels (Neuman *et al.*, 1994), we can assume that insulin treatment, by reducing blood glucose levels, decreases concentrations of circulating Amadori products, thus restoring glomerular function. In conclusion, we propose that circulating glycated albumin, already present as early as upon ten days of hyperglycemia, could enhance glomerular permeability at very early stages and could potentially lead to glomerular injuries.

### *Acknowledgments*

The authors acknowledge Gaetan Mayer and Diane Gingras for technical assistance and Jean Leveillé for photographic work.

This study was supported by a grant from the Medical Research Council of Canada.

### References

Anderson, S. and Brenner, B. M. (1988) Pathogenesis of diabetic glomerulopathy: Haemodynamic considerations. *Diabetes/Metabolism Rev.*, **4**, 163–177.

Bakala, H., Verbeke, P., Perichon, M., Corman, B. and Schaeverbeke, J. (1995) Glycation of albumin with aging and diabetes in rats: changes in its renal handling. *Mechanisms Ageing Develop.*, **78**, 63–71.

Beisswenger, P. J. and Spiro, R. G. (1970) Human glomerular basement membrane: Chemical alteration in diabetes mellitus. *Science*, **168**, 596–598.

Bendayan, M. (1985) Alteration in the distribution of type IV collagen in glomerular basal lamina in diabetic rats as revealed by immunocytochemistry and morphometrical approach. *Diabetologia*, **28**, 373–378.

Bendayan, M. (1998) Immunocytochemical detection of advanced glycated end products in rat renal tissue as function of age and diabetes. *Kidney Int.*, **54**, 438–447.

Bendayan, M. (1995) Colloidal gold post-embedding immunocytochemistry. *Prog. Histochem. Cytochem.*, **29**, 1–163.

Bendayan, M. and Londoño, I. (1996) Reabsorption of native and glycated albumin by renal proximal tubular epithelial cells. *Am. J. Physiol.*, **271**, F261–F268.

Bendayan, M. and Rasio, E. A. (1996) Transport of insulin and albumin by the microvascular endothelium of rete mirabile. *J. Cell. Sci.*, **109**, 1857–1864.

Bendayan, M., Gingras, D. and Charest, P. (1986) Distribution of endogenous albumin in the glomerular wall of streptozotocin-induced diabetic rats as revealed by high-resolution immunocytochemistr. *Diabetologia*. **29**, 868–875.

Brenner, B. M., Hostetter, T. H. and Humes, H. D. (1978a) Molecular basis of proteinuria of glomerular origin. *N. Engl. J. Med.*, **2989**, 826–833.

Brenner, B. M., Hostetter, T. H. and Humes, H. D. (1978b) Glomerular permselectivity: Barrier function based on discrimination of molecular size and charge. *Am. J. Physiol.*, **234**, F455–F460.

Bunn, H. F., Gabbay, K. H. and Gallop, P. M. (1978) The glycosylation of hemoglobin: Relevance to diabetes mellitus. *Science*, **200**, 21–27.

Castellino, P., Shohat, J. and DeFronzo, R. A. (1990) Hyperfiltration and diabetic nephropathy: Is it the beginning? or is it the end? *Semin. Nephrol.*, **10**, 228–241.

Caufield, J. P. and Farquhar, M. G. (1974) The permeability of glomerular capillaries to graded dextrans. Identification of the basement membrane of capillaries as the primary filtration barrier. *J. Cell Biol.*, **63**, 883–903.

Chiarelli, F., Verrotti, A., Mohn, A. and Morgese, G. (1997) The importance of microalbuminuria as an indicator of incipient diabetic nephropathy: Therapeutic implications. *Ann. Med.*, **29**, 439–445.

Chistiansen, J. S., Gammelgaard, J., Frandsen, M. and Parving, H. H. (1981) Increased kidney size, glomerular filtration rate and renal plasma flow in short-term insulin-dependent diabetics. *Diabetologia*, **20**, 451–456.

Cohen, M. P. and Ziyadeh, F. N. (1994) Role of Amadori-modified nonenzymatically glycated serum proteins in the pathogenesis of diabetic nephropathy. *J. Am. Soc. Nephr.*, **7**, 183–190.

Cohen, M. P., Urdanivia, E., Surma, M. L. and Wu, V. Y. (1980) Increased glycosylation of glomerular basement membrane collagen in diabetes. *Biochem. Biophys. Res. Commun.*, **95**, 765–769.

Cohen, M. P., Surma, M. L. and Wu, V. Y. (1982) *In vivo* biosynthesis and turnover of glomerular basement membrane in diabetes. *Am. J. Physiol.*, **242**, F385–F389.

Cotran, R. S. and Karnovsky, M. J. (1967) Vascular leakage induced by horseradish peroxidase in rat, *Proc. Soc. Exp. Biol. Med.*, **126**, 557–561.

Daniels, B. S. and Hausser, E. B. (1992) Glycation of albumin, not glomerular basement membrane, alter permeability in an *in vitro* model. *Diabetes*, **41**, 1415–1421.

Day, J. F., Ingelbretsen, C. G., Ingelbretsen, W. R., Baynes, J. and Thorpe, S. R. (1980) Nonenzymatic glucosylation of serum proteins and hemoglobin: Response to changes in blood glucose levels in diabetic rats. *Diabetes*, **29**, 524–527.

Deckert, T., Feldt-Rasmussen, B., Djurup, R. and Deckert, M. (1988) Glomerular size and charge selectivity in insulin-dependent diabetes mellitus. *Kidney Int.*, **33**, 100–106.

Desjardins, M. and Bendayan, M. (1990) Ultrastructural distribution of glomerular basement membrane components in experimental diabetes. *Diabetes Research*, **14**, 65–73.

Ditzel, J. and Junker, K. (1973) Abnormal glomerular filtration rate, renal plasma flow and renal protein excretion in recent and short-term diabetes. *Br. Med. J.*, **II**, 13–19.

Dolhofer, R. and Weiland, O. H. (1980) Increased glycosylation of serum albumin in diabetes mellitus. *Diabetes*, **29**, 417–422.

Farquhar, M. G., Hopper, J. and Moon, H. D. (1959) Diabetic glomerulosclerosis; electron and light microscopic studies. *Am. J. Pathol.*, **35**, 721–753.

Farquhar, M. G., Wissing, S. L. and Palade, G. E. (1961) Glomerular permeability I. Ferritin transfer across the normal glomerular capillary wall. *J. Exp. Med.*, **113**, 47–66.

Fukui, M., Nakamura, T., Ebihara, I., Shirato, I., Tomino, Y. and Koide, H. (1992) ECM gene expression and its modulation by insulin in diabetic rats. *Diabetes*, **41**, 1520–1527.

Garlick, R. L., Bunn, H. F. and Spiro, R. G. (1988) Nonenzymatic glycation of basement membranes from human glomeruli and bovine sources. *Diabetes*, **37**, 1144–1150.

Ghiggeri, G. M., Candiano, G., Delfino, G., Bianchini, F. and Queirolo, C. (1984) Glycosyl albumin and diabetic microalbuminuria: Demonstration of an altered renal handling. *Kidney Int.*, **25**, 565–570.

Ghitescu, L. and Bendayan, M. (1992) Transendothelial transport of serum albumin: A quantitative immunocytochemical study. *J. Cell. Biol.*, **117**, 745–755.

Graham, R. C. and Karnovsky, M. J. (1966) Glomerular permeability: Ultrastructural cytochemical studies using peroxidase as protein tracers. *J. Exp. Med.*, **124**, 1123–1134.

Guthrow, C. E., Morris, M. A., Day, J. F., Thorpe, S. R. and Baynes, J. W. (1979) Enhanced nonenzymatic glucosylation of human serum albumin in diabetic mellitus. *Proc. Natl. Acad. Sci. USA*, **76**, 4248–4261.

Hardwicke, J. and Squire, J. R. (1955) The relationship between plasma albumin concentration and protein excretion in patients with proteinuria. *Clin. Sci.*, **14**, 509–530.

Higgins, P. J. and Bunn, H. F. (1981) Kinetic analysis of nonenzymatic glycosylation of hemoglobin. *J. Biol. Chem.*, **256**, 5204–5208.

Hirose, K., Østerby, R., Nozawa, M. and Gundersen, H. J. G. (1982) Development of glomerular lesions in experimental long-term diabetes in the rat. *Kidney Int.*, **21**, 689–695.

Hostetter, T. H. (1986) Diabetic nephropathy. In: Brenner, B. M. and Rector, F. C. (Eds.). *The Kidney*, Philadelphia: (Ardomore Medical Books), p. 1377.

Hostetter, T. H., Troy, J. L. and Brenner, B. M. (1981) Glomerular hemodynamics in experimental diabetes mellitus. *Kidney. Int.*, **19**, 410–415.

Inoue, S. and Bendayan, M. (1995) High-resolution ultrastructural study of the rat glomerular basement membrane in long-term experimental diabetes. *Ultrastruct. Pathol.*, **19**, 175–185.

Kanwar, Y. S., Rosenzweig, L. J., Linker, A. and Jakubowski, M. L. (1983) Decreased de novo synthesis of glomerular proteoglycans in diabetes: Biochemical and autoradiographic evidence. *Proc. Natl. Acad. Sci.*, **80**, 2272–2275.

Laliberté, F., Sapin, C., Belair, M. F., Druet, P. and Bariety, J. (1978) The localization of the filtration barrier in normal rat glomeruli by ultrastructural immunoperoxidase techniques. *Biol. Cell*, **31**, 15–26.

Layton, G. J. and Jerums, G. (1988) Effect of glycation of albumin on its renal clearance in normal and diabetic rats. *Kidney Int.*, **33**, 673–676.

Londoño, I., Ghitescu, L. and Bendayan, M. (1995) Glomerular handling of circulating glycated albumin in the normal mouse kidney. *Am. J. Physiol.*, **268**, F913–F921.

McFarland, K. F., Catalano, E. W., Day, J. F., Thorpe, S. R. and Baynes, J. W. (1979) Nonenzymatic glycosylation of serum proteins in diabetes mellitus. *Diabetes*, **28**, 1011–1014.

McLean, I. W. and Nakane, P. K. (1974) Periodate-lysine-paraformaldehyde fixative. A new fixative for immunoelectron microscopy. *J. Histochem. Cytochem.*, **22**, 1077–1083.

Moriya, T., Nakazawa, K., Ittoh, N., Shigematsu, H., Okada, N., Aizawa, T., Yamada, T. and Yajima, Y. (1993) Loss of glomerular anionic sites and the development of albuminuria in rats with streptozotocin-induced diabetes. *Nephron*, **65**, 444–448.

Neuman, R. G., Hud, E. and Cohen, M. P. (1994) Glycated albumin: A marker of glycaemic status in rats with experimental diabetes. *Laboratory Animals*, **28**, 63–69.

Østerby, R. (1986) Structural changes in the diabetic kidney. *Clin. Endocrinol. Metab.*, **15**, 733–751.

Pappenheimer, J. R. (1953) Passage of molecules through capillary walls. *Physiol. Rev.*, **33**, 387–423.

Park, I. S., Kiyomoto, H., Abboud, S. L. and Abboud, H. E. (1997) Expression of transforming growth factor-beta and type IV collagen in early streptozotocin-induced diabetes. *Diabetes*, **46**, 473–480.

Pathasarathy, N. and Spiro, R. G. (1982) Effect of diabetes on the glycosaminoglycan component of the human glomerular basement membrane. *Diabetes*, **31**, 738–741.

Rohrbabach, D. H., Wagner, C. W., Star, V. L., Martin, G. R., Brown, K. S. and Yoon, J. W. (1983) Reduced synthesis of basement membrane heparan sulfate proteoglycans in diabetic mice. *J. Biol. Chem.*, **258**, 11672–11677.

Ryan, G. B. and Karnovsky, M. J. (1976) Distribution of endogenous albumin in the rat glomerular: Role of haemodynamic factors in glomerular barrier function. *Kidney Int.*, **9**, 36–45.

Sabbatini, M., Sansone, G., Uccello, F., Giliberti, A., Conte, G. and Andeucci, V. E. (1992) Early glycosylation products induce glomerular hyperfiltration in normal rats. *Kidney Int.*, **42**, 875–881.

Sampietro, T., Colantuoni, A., Lenzy, S., Bertuglia, S., Bionda, A. and Donato, L. (1987) Increased permeability of hamster microcirculation to glycosylated albumin. *The Lancet*, **31**, 994–996.

Schleicher, E. D. and Olgemöller, B. (1992) Glomerular changes in diabetes mellitus. *Eur. J. Clin. Chem. Clin. Biochem.*, **30**, 635–640.

Shaklai, N., Garlick, R. L. and Bunn, H. F. (1984) Nonenzymatic glycosylation of human serum albumin alters its conformation and function. *J. Biol. Chem.*, **259**, 3812–3817.

Shima, K., Shi, K. and Noma, Y. (1991) High performance liquid chromatographic assay of rat serum glycated albumin. *Diabetes*, **40** (suppl. 1), 205A (abstract).

Simionescu, N. (1979) Enzymatic tracers in the study of vascular permeability. *J. Histochem. Cytochem.*, **27**, 1120–1130.

Suzuki, D., Miyazaki, M., Jinde, K., Koji, T., Yagame, M., Endoh, M., Nomoto, Y. and Sakai, H. (1997) *In situ* hybridization studies of matrix metalloproteinase-3 tissue inhibitor of metalloproteinase-1 and type IV collagen in diabetic nephropathy. *Kidney Int.*, **52**, 111–119.

Trueb, B., Fluckiger, R. and Winterhalter, K. H. (1984) Nonenzymatic glycosylation of basement membrane collagen in diabetes mellitus. *Coll. Rel. Res.*, **4**, 239–251.

van den Born, J., van Kraats, A. A., Bakker, M. A. H., Assmann, K. J. M., van den Heuvel, L. P. W. J., Veerkamp, J. H. and Berden, J. H. M. (1995) Selective proteinuria in diabetic nephropathy in the rat is associated with a relative decrease in glomerular basement membrane heparan sulphate. *Diabetologia*, **38**, 161–172.

Viberti, G. C. and Wiseman, M. J. (1986) The kidney in diabetes: Significance of the early abnormalities. *Clin. Endocrinol. Metab.*, **15**, 753–782.

Viberti, G. D., MacKintosh, D. and Keen, H. (1983) Determinants of the penetration of proteins through the glomerular barrier in insulin-dependent diabetes mellitus. *Diabetes*, **32** (suppl. 2), 92–95.

Villaschi, S., Johns, L., Cirigliano, M. and Pietra, G. G. (1986) Binding and uptake of native glycosylated albumin-gold complexes in perfused rat lungs. *Microvascular Research*, **32**, 190–100.

Wieland, O. H. (1983) Protein modification by non enzymatic glucosylation: Possible role in the development of diabetic complications. *Mol. Cell. Endocrinol.*, **29**, 125–131.

Williams, S. K., Devenny, J. J. and Bitensky, M. W. (1981) Micropinocytic injestion of glycosylated albumin by isolated microvessels. Possible role in pathogenesis of diabetic microangiopathy. *Proc. Natl. Acad. Sci., USA*, **178**, 2393–2397.

Wu, K., Setty, S., Mauer, S. M., Killen, P., Nagase, H., Michael, A. F. and Tsilibary, E. C. (1997) Altered kidney matrix gene expression in early stages of experimental diabetes. *Acta. Anat.*, **158**, 155–165.

Yang, C. W., Hattori, M., Vlassara, H., He, C. J., Carome, M. A., Yamato, E., Elliot, S., Striker, G. E. and Striker, L. J. (1995) Overexpression of transforming growth factor-beta 1 mRNA is associated with up-regulation of glomerular tenascin and laminin gene expression in nonobese diabetic mice. *J. Am. Soc. Nephrol.*, **5**, 1610–1617.

Ziyadeh, F. N. and Cohen, M. P. (1993) Effects of glycated albumin on mesangial cells: Evidence for a role in diabetic nephropathy. *Mol. Cell Biochem.*, **125**, 19–25.

